# Use of the exoscope in neurosurgery

**DOI:** 10.3171/2023.10.FOCVID23188

**Published:** 2024-01-01

**Authors:** Constantinos G. Hadjipanayis, Libby K. Infinger, Martin Lehecka, Gustavo Pradilla, Gabriel Zada

**Affiliations:** 1 Department of Neurological Surgery, Center for Image-Guided Neurosurgery, University of Pittsburgh Medical Center, Pittsburgh, Pennsylvania;; 2 Department of Neurosurgery, Medical University of South Carolina, Charleston, South Carolina;; 3 Department of Neurosurgery, University of Helsinki, Finland;; 4 Department of Neurosurgery, Emory University, Atlanta, Georgia;; 5 Department of Neurosurgery, University of Southern California, Los Angeles, California

Optimal intraoperative visualization has been the cornerstone for successful completion of cranial, spinal, and peripheral nerve neurosurgery. The conventional operating microscope has been the most important advance in neurosurgery since its introduction in 1957. However, limitations of the current binocular operating microscope include its magnification, illumination, short field depth requiring frequent manipulation, and confocal view, as well as challenging surgeon ergonomics. The recent advent of the digital surgical exoscope has provided neurosurgeons with a high-definition and magnified 3D panoramic view on a heads-up display that can be used for performing surgery. The exoscope provides a longer working distance, greater magnification, and a field depth that permits neurosurgery without surgeon obstruction. LED illumination by the exoscope permits reduction in thermal damage and tissue glare that can occur with the conventional microscope. The exoscope camera system is positioned alongside the neurosurgeon with use of an independent arm to allow delineation of both normal and pathologic conditions affecting the central nervous system. Surgeon ergonomics are improved with use of the exoscope, promoting a more relaxed posture and alleviating the neck and back strain associated with the extreme head movements when using the binocular operating microscope for visualization of deep structures. The surgeon remains upright in a neutral position, minimizing back or neck tilt, as well as strain on the neck or back. The exoscope can also be used to perform fluorescence-guided surgery with appropriate light filters that include 5-aminolevulinic acid. This issue of *Neurosurgical Focus: Video* highlights use of the exoscope in cerebrovascular, neuro-oncologic, pediatric, peripheral nerve, and spinal neurosurgery. Novel and innovative video case presentations have been collected in this issue that include different exoscope technology platforms.

## Disclosures

Dr. Hadjipanayis reported personal fees from Synaptive Medical (manufacturer of the MODUS X exoscope), Stryker Corp., Hemerion Therapeutics, and Integra outside the submitted work. Dr. Zada reported consulting fees from Integra outside the submitted work.

